# *Nuurcala obesa* sp. n. (Blattida, Caloblattinidae) from the Lower Cretaceous Yixian Formation in Liaoning Province, China

**DOI:** 10.3897/zookeys.318.5514

**Published:** 2013-07-23

**Authors:** Chongda Wang, Dong Ren

**Affiliations:** 1 College of Life Sciences, Capital Normal University, 105 Xisanhuanbeilu, Haidian District, Beijing 100048, China

**Keywords:** Fossil cockroach, new species, Huangbanjigou, wing venation, colouration

## Abstract

*Nuurcala obesa* **sp. n.**, in the cockroach family Caloblattinidae, is described from the Lower Cretaceous Yixian Formation (Liaoning Province, China) based on a combination of differential characters of fore- and hind wing venation, colouration and body structures. Systematically, *Nuurcala* (Vršanský, 2003), known from the Cretaceous sediments of Bon Tsagaan and Khurilt, Mongolia, is closely related to other genera of Caloblattinidae known from the Jurassic and Cretaceous localities in other parts of Asia.

## Introduction

Caloblattinidae, a large extinct family of cockroaches, was erected by Vršanský and Ansorge, it consists of over 50 genera comprising nearly 200 described species distributed in Argentina, Australia, Brazil, Burma, China, England, France, Germany, Greenland, Japan, Kazakhstan, Kirgizstan, Mongolia, Russia, Spain, Switzerland, and South Africa during Middle Triassic to Late Cretaceous (Vršanský 2000, 2008b). Up to now, the Caloblattinidae is presently under review and a number of taxa (both genera and species) are still waiting reassessment (Vršanský 2007), most of them were transferred from the taxonomical “waste basket” of the family Mesoblattinidae (Vršanský 2000). Caloblattinids probably originated from Phyloblattidae in the Early Triassic, representing part of the cockroach lineage leading from the older ancestors toward living cockroach taxa (Vršanský et al. 2002, 2003b). This family is distinguished by its large body, fairly long ovipositors in females, and both wings with dark colouration and veins multiple-branched (Vršanský 2000). Amber specimens of caloblattinids are extraordinary rare probably due to their large size resulting in lower probability of amber inclusion (Vršanský 2009).

Caloblattinidae was the dominant family from Upper Jurassic in Karatau, South Kazakhstan (Vishniakova 1968, 1973). The Raphidiomimidae originated from the Caloblattinidae (which is presumed to be paraphyletic family with respect to Raphidiomimidae, Liberiblattinidae and some other extinct families) (Vršanský 2003a). Both Caloblattinidae and Raphidiomimidae share strong synapomorphies such as wide abdominal segments, long palps, elongated wings with apparent intercalaries, diagonal fold in the forewing, hind wing with many reticulations in CuA-CuP space, R with R1 and RS abundantly branched, M weakly branched, CuA secondarily branched (Liang et al. 2009). We have collected about 1500 fossil cockroaches in sediments of the Early Cretaceous of Yixian Formation, most of them belong to Blattulidae (Wang et al. 2007a, b). However, so far, only 2 species of Caloblattinidae have been described: *Rhipidoblattina laternoforma* (Lin, 1978), *Euryblattula beibiaoensis* (Wang, 1987). This indicates in the Early Cretaceous, Caloblattinidae were not dominant in the Yixian Formation.

The strata of the Yixian Formation represent mainly lacustrine sediments intercalated with volcaniclastics, which contains a large number of Jehol Biota fossils, such as well-preserved dinosaurs, primitive birds, early mammals, fishes, ostracods, plants and abundant insects (Sun et al. 1998, Hou et al. 1999, Ding et al. 2001, Ren et al. 2010). Palaeobotanical data, including spores, pollen and plants described, indicate warm and moist climate (Ding et al. 2001). Zhang et al. (2004) and Xing et al. (2005) respectively base on isotope data and abundant statistical analysis of fossils data, coming to the consistent opinion that the age of Yixian Formation is determined as Early Cretaceous. And this opinion has been accepted widely (Swisher et al. 1999, Lu 2000, Zhou et al. 2003, Franz et al. 2007). Here we consider the age of Yixian Formation as the Early Cretaceous (about 125 Ma).

## Material and methods

The four specimens were collected from the Yixian Formation, Huangbanjigou, Chaomidian Village, Beipiao City, Liaoning Province, China. All type specimens are deposited in the fossil insect collection of the Key Laboratory of Insect Evolution & Environmental Changes, Capital Normal University, Beijing, China. They were examined with a Leica MZ 12.5 dissecting microscope and illustrated with the aid of a drawing tube attached to the microscope. Line drawings were made with Photoshop CS 3.0 graphic software. Photographs of fossils were taken by a MZ12.5 dissecting microscope (Leica, Wetzlar, Germany), either dry or with alcohol.

The venation nomenclature used in this paper is based on the interpretation of Comstock and Needham (1898), followed also by Vishniakova (1964) and Vršanský (1997 and later). Abbreviations used: RFW - Right forewing; LFW - Left forewing; HW - Hind wing; Sc - Subcosta; R - Radius; Rs - Radius Sector; M - Media; Cu - Cubitus (A - anterior, P - posterior); A - Anal veins; Ant - Antenna. PIN – Paleontological Institute, Russian Academy of Sciences, Moscow, Russia. CNU – Capital Normal University, Beijing. PCMAS – Paleontological Center of Mongolian Academy of Sciences.

## Systematic palaeontology

### Order Blattida Latreille, 1810 (= Blattaria Latreille, 1810; = Blattodea Brunner von Wattenwyl, 1882)
Superfamily Caloblattinoidea Vršanský & Ansorge, 2000
Family Caloblattinidae Vršanský & Ansorge, 2000

#### Genus Nuurcala

Vršanský, 2003

http://species-id.net/wiki/Nuurcala

##### Type species.

*Nuurcala popovi* Vršanský, 2003

##### Composition.

*Nuurcala popovi* Vršanský, 2003. Bon Tsagaan Nuur, Bed 87/8, Mongolia; Barremian or Aptian, Early Cretaceous.

*Nuurcala srneci* Vršanský, 2008. Khurilt, Bed 210/24, Mongolia, Barremian or Aptian, Early Cretaceous.

*Nuurcala* sp. (collected by expedition of PIN led by M.B. Mostovski and P. Vršanský) Baissa, Transbaikalian Russia, ?Valanginian, Early Cretaceous.

*Nuurcala* sp. (collected by D. Davaadorj, deposited in the PCMAS, undescribed) Erdenyi Ula, Mongolia, Early Cretaceous.

##### Stratigraphic and paleogeographic range of the genus.

?uppermost Jurassic; Lower - Upper Cretaceous; Asia.

#### Nuurcala obesa

Wang & Ren sp. n.

urn:lsid:zoobank.org:act:1009795B-D0EA-41D4-A356-B3FEA7515871

http://species-id.net/wiki/Nuurcala_obesa

Figs 1 [Fig F2] – 4

##### Differential diagnosis.

**Figure 1.**
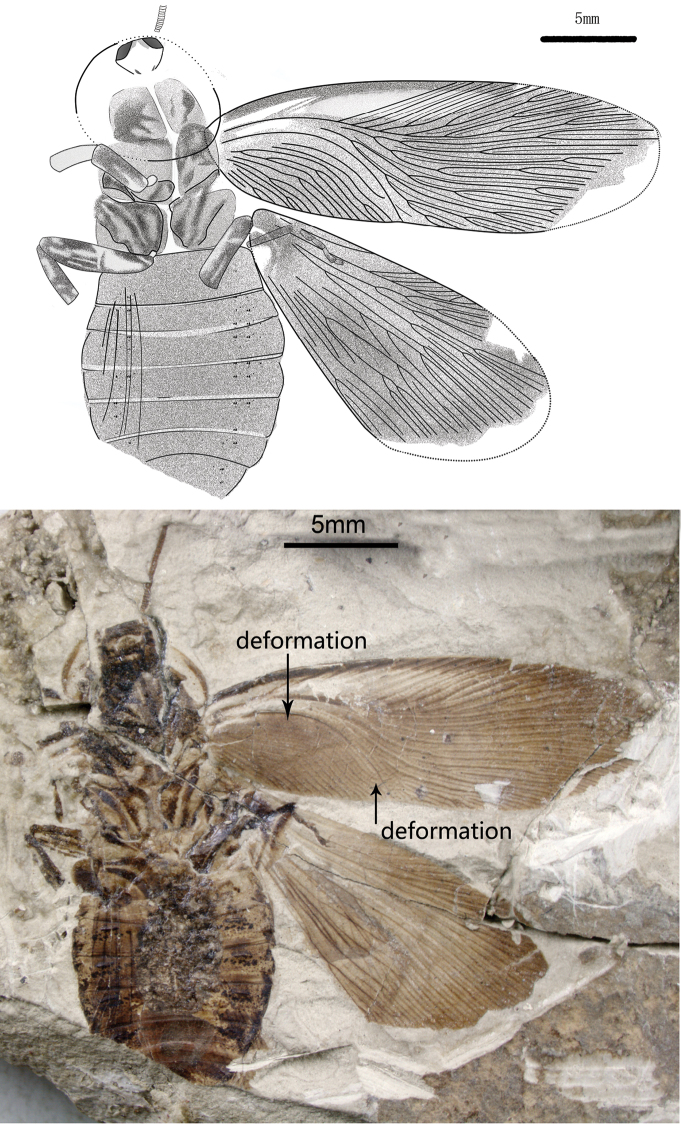
*Nuurcala obesa*, sp. n. Holotype, CNU-BLA-NN-2012055 **A** Line drawing **B** photograph. Scale bars = 5 mm.

Differs from *Nuurcala popovi* and *Nuurcala srneci* in having relatively small head, and antennal sockets conspicuous at sides, antennae long and thick; forewing with dark colouration except for R area, a dark maculae present at the edge of Sc area; hindwing with dark colouration; Legs with dark colouration, femora thick, tarsus with five segments and a claw (Fig. 1).

##### Description.

Holotype (Fig. 1): body medium to large size, length about 23.8 mm as preserved, total estimated length is 25.2 mm, and width 9.9 mm; Abdomen with 6–7 visible segments. head small, length 1.8 mm, and width 2.5 mm, antennal sockets conspicuous at sides. Pronotum, shield-like, vaulted, transversal, simple symmetrical zonal colouration at the margin, length 6.2 mm, width 6.9 mm. Forewings: length 22 mm, width 6 mm; one dark maculae present at the edge of Sc area, dark colouration with pale area in R; 55 veins at margin; intercalaries thick, all over wing surface; Sc 3 branches, shorter than clavus; R sigmoidal 15 branches, with undifferentiated Rs, reaching the anterior wing margin; M slightly curved with 9 branches, most posterior branches of M reaching wing apex; CuA slightly curved to posterior wing margin and 10 branches; CuP curved and simple; clavus long, more than a third of the wing’s length; A with tertiary branches. Hind wing (17 mm long as preserved vs. 22 mm long for forewing) with branched Sc; both R1 and RS about 9 veins; M 4 branches; Cu (±9) with additional blind branches that may reticulate, fan-like pleating present visible on forewing. Legs with dark colouration, femora thick, about 2 times as long as tibia, tarsus with five segments and a claw, spines obscure.

**Figure 2. F2:**
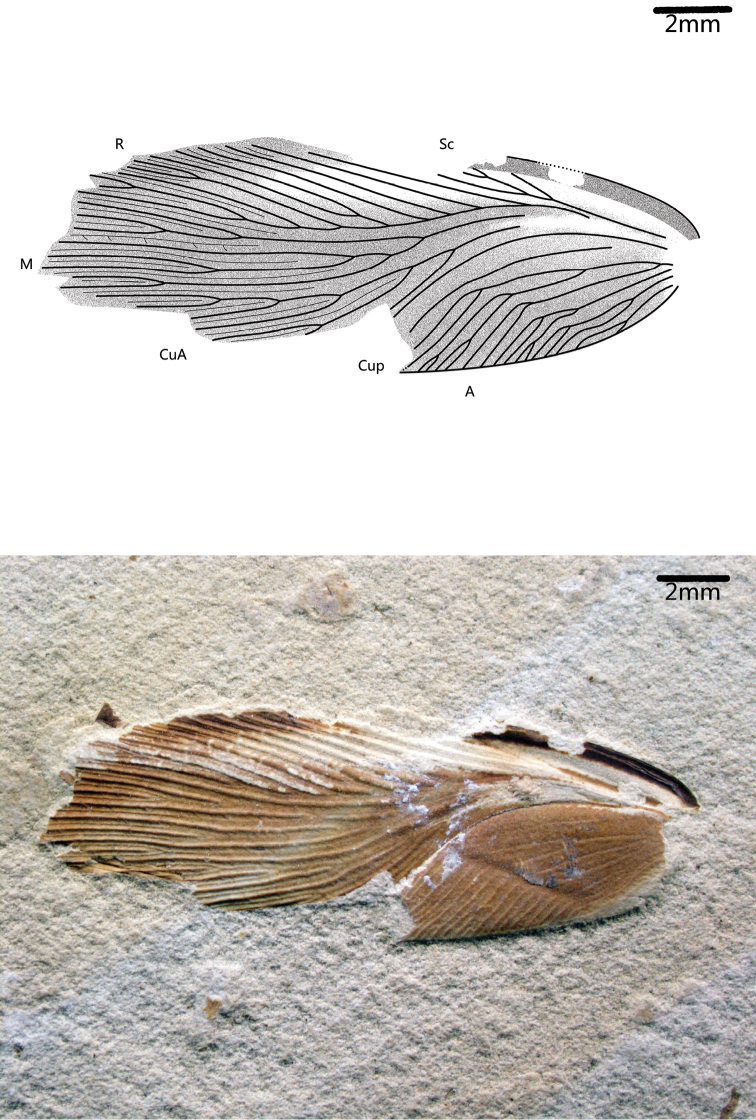
*Nuurcala obesa*, sp. n. Paratype, CNU-BLA-NN-2012056 **A** Line drawing **B** photograph. Scale bars = 2 mm.

**Figure 3.**
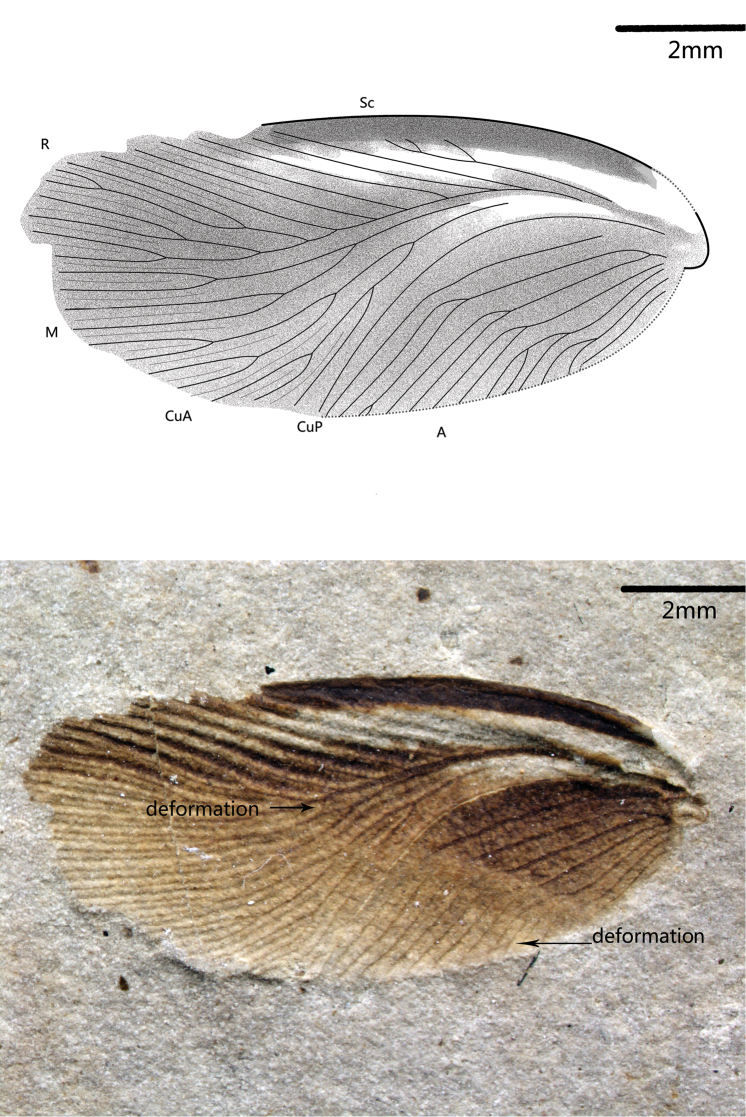
*Nuurcala obesa*, sp. n. Paratype, CNU-BLA-NN-2012057 **A** Line drawing **B** photograph. Scale bars = 2 mm.

**Figure 4.**
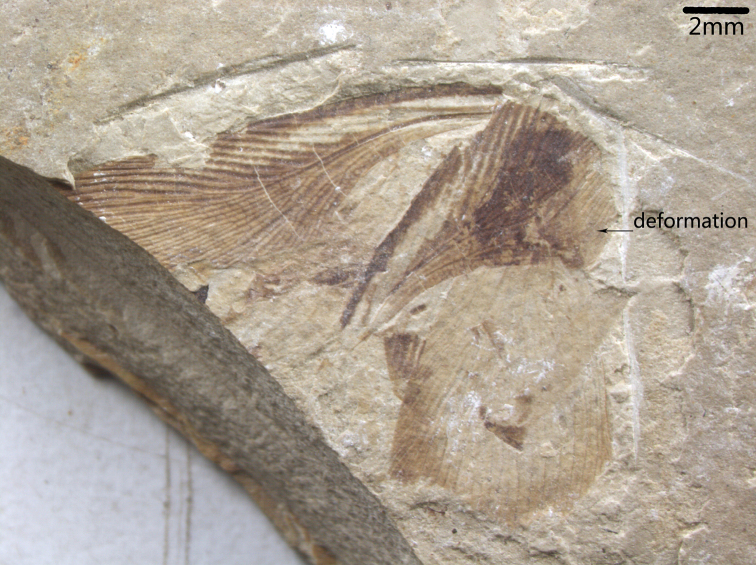
*Nuurcala obesa*, sp. n. Paratype, CNU-BLA-NN-2012058; photograph. Scale bar = 2 mm.

Paratypes (Figs 2–4): only forewing preserved, length range about 11.5–19 mm, width range about 5.2–5.7 mm; 42–48 veins at margin; Sc 3-5 branches, R sigmoidal 12–15 branches, M slightly curved and 4–9 branches, CuA 7–10 branches.

##### Materials.

Holotype, partially preserved specimen with both fore- and hind wings and a body, CNU-BLA-NN-2012055 (Fig. 1). Paratypes (three isolated forewings): CNU-BLA-NN-2012056 (Fig. 2), CNU-BLA-NN-2011057 (Fig. 3), CNU-BLA-NN-2011058 (Fig. 4).

##### Type locality and horizon.

Yixian Formation; Early Cretaceous; Huangbanjigou, Chaomidian Village, near Beipiao City, Liaoning Province, China.

##### Etymology.

The specific name is derived from the Latin word “obesus”, (meaning “fat”), refer to the relatively large abdomen.

## Discussion

We consider the present taxon belonging to the genus *Nuurcala* based on the following features: Body medium to large size, both wings have dark markings, and forewing with characteristic colouration pattern. The four specimens vary in size significantly (wing length from 11.5 to 22 mm), and the size range of this species supports that Caloblattinidae are highly variable in size (Vršanský 2000), which is different from the Blattulidae (Wang et al. 2007a, b).

*Nuurcala obesa* sp. n. is similar to *Nuurcala popovi* Vršanský, 2003 in the following aspects: head hypognathous, pronotum transverse ovoid, and forewing with distinct cubital space, rather wide, but differs from *Nuurcala popovi* Vršanský, 2003 by forewings with subparallel margins and characteristic markings (dark markings with pale area in R), Sc branched, R rich, M branched, Cu veins ending prior to the apex of the wing, A branched, CuA almost straight, and then curved to posterior wing margin, anal area wide.

*Nuurcala obesa* sp. n. differs from *Nuurcala srneci* Vršanský, 2008 by the latter having much bigger head and the veins less numerous than *Nuurcala obesa*. The new speciesdiffers from *Nuurcala* sp. (PCMAS) from Erdenyi Ula, Mongolia in having a bigger pronotum (in contrast to 5.8/6.2 mm; forewing length 21 mm).

The number of forewing veins in *Nuurcala obesa* sp. n. is differs from those of previously reported *Nuurcala popovi* and *Nuurcala srneci*. For comparison, the data are listed in Table 1. The total number of forewing veins of *Nuurcala obesa* (42–55 veins) is higher than that of *Nuurcala srneci* (about 44 veins), but much less than that of *Nuurcala popovi* (54–83 veins). The number of forewing M veins of *Nuurcala obesa* (4–9 veins) is lower than those in *Nuurcala popovi* (8–17 veins) and *Nuurcala srneci* (6–11). The differences of venation further justify the erection of *Nuurcala obesa* sp. n.

**Table 1.** Variability of number of veins in forewings for three species of *Nuurcala*.

**Species**	**Number of veins in forewings**					
	**Sc**	**R**	**M**	**Cu**	**A**	**Total**
*Nuurcala obesa*	1–3	12–15	4–9	8–10	14–19	42–55
*Nuurcala popovi*	3–5	16–25	8–17	12–17	15–21	54–83
*Nuurcala srneci*	±3	13–16	6–11	8–14	5–7	±44

There are some taxa placed in other genera which show affinities to the *Nuurcala*. *Nuurcala obesa* sp. n. differs from *Rhipidoblattina hebeiensis* Hong, 1980 from the Middle Jurassic Jiulongshan Formation (Hong 1980) by the dark maculae at the edge of Sc area, different dark colouration position at forewing, and more A veins for the new species. The new species is closely similar to *Samaroblatta nitida* Lin, 1986, which has the same forewing type and the intercalaries, but differs in venation character and the dark maculae (Lin 1986). The veins of new species have more branches than *Samaroblatta rhypha* Lin, 1986 and *Summatiblatta colorata* Lin, 1986. The new species differs from *Soliblatta lampra* Lin, 1986 by the shape of forewing, the latter with a lance shaped forewing (Lin 1986). The new species differs from *Shartegoblattina colorata* Vršanský, 2005 from the Sharin-Gol in Mongolia by the characters as follows: the new species just has one dark maculae present at the edge of Sc area, but *Shartegoblattina colorata* Vršanský, 2005 with colouration dark along the fore margin, more pale in the distal part than the new species. The new species differs obviously from *Solemnia alexandri* Vršanský, 2008 from the Houtiyn-Hotgor Locality in Mongolia due to the latter forewings extremely elongated.

Yixian Formation is characterized by a high proportion of coloured species (Wang et al. 2007a, b, Wei et al. 2013). Dry habitats are rather characterized by monochromatic and pale cockroach individuals (Vršanský et al. 2009, Wei and Ren in press). The new taxon supports the notion that Yixian Formation was humid and moist.

The family Caloblattinidae, starting with its first occurrence in the Middle Triassic, was important in almost all known ecosystems and dominated from the earliest Jurassic up to the latest Early Cretaceous (Vršanský 2008b). It is enigmatic that the family Caloblattinidae was rare in the Yixian Formation. Only 4 species have been described so far (Ren et al. 1995, Hong 1983, and this study). This is in contrast to the fact that in nearly all Jurassic and Cretaceous localities, this family is dominant or co-dominant (Vršanský et al. 2002). The Cretaceous is the most dynamic period in the history of the order. The transition between the Jurassic and Cretaceous is characterised by the change in the dominant families, and by appearance of extant families in the fossil record. Caloblattinidae have been replaced by Blattellidae, Mesoblattinidae and, to a lesser extent, by Blattulidae as the dominant families (Vršanský et al. 2002). In the Yixian formation, Blattulidae were dominant, and their taxonomic analysis supports the Jurassic/Lower Cretaceous age (Wang et al. 2007a, b). During Upper Jurassic/Lower Cretaceous transition, Caloblattinidae are known mostly from the rich assemblages in Karatau, South Kazakhstan (Vishniakova 1968, 1973), and the less rich one in Argentina, Australia, Brazil, Burma, China, England, France, Germany, Greenland, Japan, Kazakhstan, Kirgizstan, Mongolia, Russia, Spain,Switzerland, and South Africa. Comparing climates of Yixian Formation and other regions, the warm and moist climate of Yixian Formation might have been more suitable for Blattulidae.

## Supplementary Material

XML Treatment for Nuurcala

XML Treatment for Nuurcala obesa

## References

[B1] Comstock JH Needham JG (1898) The wings of insects. American Naturalist 32 (376): 231-257. doi: 10.1086/276835

[B2] Chen PJ Wang QF Zhang HC Cao MZ Li WB Wu SQ Shen YB (2004) Discussion on the stratotype of Jianshangou of Yixian Formation. Science in China Series, D, Earth Sciences 34: 883-895. [In Chinese with English abstract]

[B3] Ding DH Zhang LD Guo SZ Zhang CJ Peng YD Jia B Chen SW Xing, DH (2001) The stratigraphic sequence and fossil bearing horizon of the Yixian Formation in western Liaoning, China. Geology and Resources 10 (4): 193-198.

[B4] Hong YC (1980) New genus and species of Mesoblattinidae in China. Bulletin Chi nese Acdemic Geological Science, Series VI 1 (2): 49-60. [In Chinese]

[B5] Hong YC (1983) Middle Jurassic Fossil Insects in North China. Geological Publishing House, Beijing: 26237.

[B6] Hou LH Martin LD Zhou ZH (1999) A diapsid skull in a new species of the primitive bird Confuciusornis. Nature 399: 672-682.

[B7] Li PX Cheng ZW Pang QQ (2001) The horizon and age of Confuciusornis in Beipiao, western Liaoning. Acta Geologica Sinica 75: 1-13. [In Chinese with English abstract]

[B8] Liang JH (2006) The fossil Blattaria of China – a review of present knowledge In: Acta Zootaxonomica Sinica 31: 102–108. [In Chinese]

[B9] Lin QB (1986) Early Mesozoic fossil insect from the South China. Palaeontologica Si nica Series B, No. 21 Science Press, Beijing, 28–53.

[B10] Pang QQ Li PX Tian SG Liu YQ (2002) Discovery of ostracods in the Dabeigou and Dadianzi Formations at Zhangjiagou, Luanping County, northern Hebei Province of China and new progress in the biostratigraphic boundary study. Geological Bulletin of China 21: 329–336. [In Chinese with English abstract]

[B11] Ren D Shih CK Gao TP Yao YZ Zhao YY (2010) Silent Stories–Insect Fossil Treasures from Dinosaur Era of the Northeastern China. Science Press, Beijing, 322 pp.

[B12] Ren D Lu LW Ji SA Guo ZG (1995) Faunae and Stratigraphy of Jurassic-Cretaceous in Beijing and the adjacent areas. Seismic Publishing House, Beijing, China, 222 pp. [In Chinese]

[B13] Sun GD Dilcher L Zheng SL Zhou ZK (1998) In search of the first flowers: a Jurassic angiosperm, Archaefructus, from Northeast China. Science 282 (5394): 1692-1695. doi: 10.1126/science.282.5394.1692 9831557

[B14] Smith PE Evensen NM York D Zhang MM Jin F Li JL Cumbaa S Russell DA (1995) Dates and rates in ancient lakes: 40Ar-39Ar evidence from an Early Cretaceous age for the Jehol Group, northeast China. Canadian Journal of Earth Sciences 32: 1426-1431. doi: 10.1139/e95-115

[B15] Swisher CC Wang XL Xu X Wang Y (1999) Cretaceous age for the feathered dinosaurs of Liaoning, China. Nature 400: 58-61. doi: 10.1038/21872

[B16] Vishniakova VN (1964) Additional characters of wing venation in forewings of a new Upper Jurassic cockroach. Paleontological Journal 1964 (1): 82-87. [In Russian]

[B17] Vishniakova VN (1968) New cockroaches (Insecta: Blattodea) from the Upper Jurassic of Karatau mountains. In: Rohdendorf BB (Ed). Jurassic insects from Karatau Nauka, Moscow, 55–86.

[B18] Vishniakova VN (1973) New cockroaches (Insecta: Blattodea) from the Upper Jurassic deposits of Karatau. In: Narchuk EP (Ed). Voprosy paleontologii nasekomykh. Doklady na 24-m Ezhegodnom chtenii pamyati N.A.Kholodkovskogo, 1971. [Problems of the Insect Palaeontology. Lectures on the XXIV Annual Readings in Memory of N.A. Kholodkovsky (1–2 April, 1971)]. Nauka, Leningrad: 64-77. [In Russian]

[B19] Vršanský P (1997) Piniblattella gen. nov. - the most ancient genus of the family Blattellidae (Blattodea) from the Lower Cretaceous of Siberia. Entomol. Probl. 28 (1): 67-79.

[B20] Vršanský P (2000) Decreasing variability-from the Carboniferous to the Present! (Validated on independent Iineages of Blattaria). Paleontological Journal 34 (3): 374-379.

[B21] Vršanský P Vishniakova, VN Rasnitsyn AP (2002) Order Blattida Latreille, 1810. In: Rasnitsyn AP Quicke DLJ (Eds). 2002 History of Insects. Dodrecht etc.: Kluwer Academic Publishers: 263-270.

[B22] Vršanský P (2003a) Phyloblatta grimaldii sp. nov. – a new Triassic cockroach (Insecta: Blattaria) from Virginia. Entomological Problems 33(1–2)): 51-53.

[B23] Vršanský P (2003b) Unique assemblage of Dictyoptera (Insecta—Blattaria, Mantodea, Isoptera) from the Lower Cretaceous of Bon Tsagaan Nuur in Mongolia. Entomological Problems 33: 119-151.

[B24] Vršanský P (2005) Lower Cretaceous cockroaches and mantids (Insecta: Blattaria, Mantodea) from the Sharin−Gol in Mongolia. Entomological Problems 35: 163-167.

[B25] Vršanský P (2007) Jumping cockroaches (Blattaria, Skokidae fam n.) from the Late Jurassic of Karatau in Kazakhstan. Biologica (Section Zoology) 62 (5): 588-592.

[B26] Vršanský P (2008a) New blattarians and a review of dictyopteran assemblages from the Lower Cretaceous of Mongolia. Acta Palaeontologica Polonica 53 (1): 129-136. doi: 10.4202/app.2008.0109

[B27] Vršanský P (2008b) Late Jurassic Cockroaches (Insecta, Blattaria) from the Houtiyn-Hotgor Locality in Mongolia. Paleontological Journal 42 (1): 36–42.

[B28] Vršanský P (2009) Albian cockroaches (Insecta, Blattida) from French amber of Archingeay. Geodiversitas 31 (1): 73-98. doi: 10.5252/g2009n1a7

[B29] Vršanský P Liang JH Ren D (2009) Advanced morphology and behaviour of extinct earwig-like cockroaches Blattida: Fuziidae). Geologica Carpathica 60 (6): 449-462.

[B30] Wang TT Liang JH Ren D (2007a) Variability of Habroblattula drepanoides gen. et. sp. nov. (Insecta: Blattaria: Blattulidae) from the Yixian Formation in Liaoning, China. Zootaxa 1443: 17-27.

[B31] Wang TT Liang JH Ren D Shi C (2007b) New Mesozoic cockroaches (Blattaria: Blattulidae) from Jehol Biota of western Liaoning in China. Ann. Zool 57 (3): 483-495.

[B32] Wang WL Zhang LJ Zheng SL Zheng YJ Zhang H Li ZT Yang FL (2004) A new study on the stratotypeand biostratigraphy of the Yixian stage in Yixian, Beipiao region, Liaoning. Establishment and study of stratotypesof the Yixian Stage. Acta Geologica Sinica 78: 433–447. [In Chinese]

[B33] Wang WL Zhang LJ Zheng SL Zheng YJ Zhang H Li ZT Yang FL (2005) The age of the Yixian Stageand the boundary of Jurassic-Cretaceous-the establishment and study of stratotypes of the Yixian Stage. Geological Review 51: 234-242. [In Chinese].

[B34] Wei DD Liang JH Ren D (2013) A new fossil genus of the Fuziidae (Insecta, Blattida) from the Middle Jurassic of Jiulongshan Formation, China. Geodiversitas 35 (2): 335-343. doi: 10.5252/g2013n2a3

[B35] Wei DD Ren D (in press) Completely preserved cockroaches of the family Mesoblattinidae from the Upper Jurassic or Lower Cretaceous Yixian Formation (Liaoning Province, Northeast China). Geologica Carpathica.

[B36] Xing DH Sun CL Sun YW Zhang LD Peng YD Chen SW (2005) New knowledge on Yixian Formation. Acta geoscientica sinica 26 (1): 25-30. [In Chinese with English abstract]

[B37] Zhang LD Jin CZ Guo SZ Zhang CJ Peng YD Chen SW Xing DH Ding QH Zheng YJ (2004) The precious fossil-bearing beds of Yixian Formation in Beipiao-Yixian area: their ages and correlation. Geology and resources 13 (4): 193-201. [In Chinese with English abstract]

[B38] Zhang JF (1985) New data of Mesozoic insect fossil from Laiyang in Shandong. Geol. Shandong 1 (2): 23-39.

[B39] Zhang ZJ Lu LW Jin YX Fang XS Hong YC (2003) Discovery of fossil insects in the Tuodian Formation, central Yunnan. Geological Bulletin of China 22: 452-455. [In Chinese with English abstract]

[B40] Zheng SL Zheng YJ Xing DH (2003) Characteristics, age and climate of late Jurassic Yixian flora from western Liaoning. Journal of Stratigraphy 27: 233-241. [In Chinese with English abstract]

[B41] Zhou ZH Barrett PM Hilton J (2003) An exceptionally preserved Lower Cretaceous ecosystem. Nature 421: 807-814. doi: 10.1038/nature01420 12594504

